# Pre-emptive Dexmedetomidine Versus Magnesium Sulfate for Postoperative Analgesia in Nasal Surgery: A Prospective Comparative Study

**DOI:** 10.7759/cureus.115498

**Published:** 2026-08-31

**Authors:** Charu Charu, Ravi Goel, Pragya Pragya, Nishi Kulshreshtha

**Affiliations:** 1 Anesthesiology and Critical Care, Batra Hospital and Medical Research Centre, New Delhi, IND; 2 Anesthesiology and Critical Care, Lady Hardinge Medical College, New Delhi, IND

**Keywords:** dexmedetomidine, magnesium sulfate, nasal surgery, postoperative pain, pre-emptive analgesia

## Abstract

Background and objective: Dexmedetomidine and magnesium sulfate are commonly used during nasal surgery to provide controlled hypotension. This study compared their effects on postoperative analgesia. The primary outcome was postoperative pain assessed using the Visual Analogue Scale (VAS). Secondary outcomes included hemodynamic parameters, time to first rescue analgesic and total rescue analgesic consumption, and patient satisfaction.

Materials and methods: This prospective, non-randomized comparative study included 75 patients with American Society of Anesthesiologists (ASA) physical status I-II undergoing nasal surgery under general anesthesia. Eligible patients were enrolled consecutively and observed across three exposure groups of 25 each, categorized according to the adjuvant selected by the treating anesthesiologist as part of routine clinical practice. Group D received a dexmedetomidine 0.5 µg/kg IV bolus administered over 10 minutes, 15 minutes before induction, followed by an infusion at 0.25 µg/kg/hour from induction until 15 minutes before the end of surgery. Group M patients received magnesium sulfate 40 mg/kg diluted in 100 mL normal saline administered IV over 15 minutes before induction, while group C received no anesthetic adjuvants and served as controls. We recorded VAS scores, hemodynamic variables, and rescue analgesics at predefined intervals up to 24 hours postoperatively.

Results and conclusion: Postoperative VAS scores were significantly lower with dexmedetomidine than with magnesium sulfate at one to two hours (0.64±0.76 vs 2.04±0.84; 1.24±0.78 vs 2.60±1.47; p <0.001), and both groups were lower than controls in the first six hours (p <0.05). Time to first rescue analgesia was longer with dexmedetomidine (285±46.63 min) than with magnesium sulfate (147.27±49.21 min; p <0.001), with lower analgesic consumption and higher patient satisfaction (p <0.001). Pre-emptive dexmedetomidine was associated with improved postoperative analgesia and reduced rescue analgesic requirement after nasal surgery.

## Introduction

Postoperative pain and discomfort remain concerns after nasal surgeries and are exacerbated by nasal packing [1]. Current anesthesia practice emphasizes opioid-free anesthesia and multimodal analgesia within Enhanced Recovery After Surgery (ERAS) protocols [2]. Pre-emptive analgesia, part of multimodal analgesia, is antinociceptive treatment initiated before a surgical stimulus to prevent central sensitization. It is defined as an antinociceptive treatment that prevents the establishment of altered central processing of afferent input from injuries. Various agents have been evaluated, including opioids, pregabalin, and other analgesic adjuvants [3,4]. Alpha‑2(α2) agonists are an attractive choice for providing intraoperative and postoperative analgesia. Dexmedetomidine, an α2-agonist, provides sedation, analgesia, and sympatholysis, while magnesium sulfate, via N-methyl-D-aspartate (NMDA) antagonism and calcium channel blockade, offers analgesic and opioid-sparing effects [5-9]. 

Both dexmedetomidine and magnesium sulfate have been used to facilitate controlled hypotension during nasal surgery, and the treating anesthesiologist may select either agent [10-14]. The variation in perioperative adjuvant choice in routine clinical practice provides an opportunity to compare analgesic and hemodynamic effects without investigator-directed treatment allocation. Therefore, we conducted a prospective observational study comparing pre-emptive dexmedetomidine and magnesium sulfate with standard care for postoperative analgesia, hemodynamic parameters, and rescue analgesic requirements in patients undergoing nasal surgery under general anesthesia. 

## Materials and methods

Study design and setting

This prospective, comparative, observational study was conducted in the Department of Anesthesiology at Batra Hospital and Medical Research Center (New Delhi, IND). Patient enrollment and data collection were conducted over 24 months, from January 2019 to December 2020. The study evaluated the effects of dexmedetomidine and magnesium sulfate on postoperative analgesia in patients scheduled for elective nasal surgeries lasting up to 90 minutes under general anesthesia. The patients were categorized into one of three groups: (1) pre-emptive dexmedetomidine 0.5 µg/kg IV bolus administered over 10 minutes, 15 minutes before induction, followed by an infusion at 0.25 µg/kg/hour from induction until 15 minutes before the end of surgery (group D); (2) pre-emptive magnesium sulfate 40 mg/kg diluted in 100 mL normal saline administered IV over 15 minutes before induction (group M); or (3) standard general anesthesia without pre-emptive analgesic adjuvant (group C), according to the treating anesthesiologist's routine clinical practice at pre-anesthetic evaluation. The choice of adjuvant was not directed or controlled by the investigators. A blind assessor observed patients postoperatively. Pain was assessed using the Visual Analogue Scale (VAS) [15] at 30 minutes, the first to sixth hour, the 12th hour, and 24 hours with hemodynamic parameters, including heart rate (HR) and mean arterial pressure (MAP), adverse effects (nausea, vomiting, drowsiness), time to first rescue analgesia, total analgesic consumption, and overall satisfaction on a 5-point Likert scale (excellent = 5; good = 4; fair = 3; poor = 2; bad = 1).

Ethics committee approval

The Scientific Research and Ethical Review Committee (SRERC) approved the study (approval no. SRERC/2019/21/14) on 28/01/2019 before the study began. Patients provided written informed consent after a detailed explanation of the observational procedures, in accordance with the Declaration of Helsinki. The study was registered with the Clinical Trials Registry-India on 25/03/2019 (CTRI/2019/03/018255), in accordance with the institutional protocol. As an observational study, investigators did not assign treatment; adjuvant choice followed routine clinical practice, and the resulting treatment groups were subsequently compared. 

Inclusion and exclusion criteria

Patients with the following criteria were included in the study: aged between 18 and 60 years, both male and female, with a BMI of 18.5 to 24.9 kg/m², American Society of Anesthesiologists (ASA) physical status grade I and II, and the capability to comprehend the VAS planned for surgeries such as functional endoscopic sinus surgery (FESS), FESS with septoplasty, FESS with grommet insertion, septorhinoplasty, and rhinoplasty. Patients were excluded if they were younger than 18 years or older than 60 years; had an ASA physical status of III or IV; declined to provide informed consent; had a BMI >24.9 kg/m²; had cardiorespiratory, renal, hepatic, neurological, bleeding, or neuromuscular disorders; had known allergies to any of the study drugs; were pregnant or lactating; had a history of alcohol or drug abuse; or were receiving calcium channel blockers. 

Sample size calculation and method

The sample size estimation is based on the effects of two drugs, namely pre-emptive dexmedetomidine and magnesium sulfate, on postoperative pain relief after nasal surgeries under general anesthesia. This is based on previously published studies by Kim et al. and Asadollah et al. [16,17]: with a difference of one in patients’ mean VAS score between the two groups, an effect size of 1.0 at a two-sided alpha of 0.05, a CI of 95%, and a power of 90%, the sample size was calculated to be 21 per group. To account for potential dropouts, we included 25 participants in each of the three groups, for a total sample size of 75. We used consecutive sampling to enroll eligible patients for elective nasal surgery in the order they arrived. They were categorized into exposure groups according to the adjuvant used in their anesthetic care until the target sample size for each group was met.

Data collection

All enrolled patients followed standard fasting guidelines, and written informed consent was obtained; they were familiarized with the 10-point VAS. The standard ASA monitoring was applied. After gaining IV access, 1 mg of midazolam was administered. In group D, dexmedetomidine was administered as an initial bolus followed by a continuous infusion, which was discontinued 15 minutes before the end of surgery. Induction was performed with fentanyl 2 µg/kg and propofol 2 mg/kg IV. Vecuronium 0.1 mg/kg facilitated endotracheal intubation. In group M, magnesium sulfate was administered intravenously before induction of anesthesia. Anesthesia was maintained with isoflurane in oxygen and nitrous oxide, maintaining end-tidal carbon dioxide between 35 mmHg and 40 mmHg. Supplemental fentanyl and vecuronium boluses were administered as required. All patients received 1 g of paracetamol IV intraoperatively.

Bradycardia (pulse rate ≤50/min) and hypotension (fall in mean arterial pressure >20% of baseline), if they occurred, were treated with atropine 0.6 mg and ephedrine 6 mg IV, respectively. Neuromuscular blockade was reversed with neostigmine 0.05 mg/kg and glycopyrrolate 0.01 mg/kg. We administered ondansetron 4 mg IV for postoperative nausea and vomiting (PONV) prophylaxis. Patients were monitored in the post-anesthesia care unit (PACU) for two hours before being transferred to the ward. An investigator blinded to group assignment assessed outcomes. Pain was assessed using a VAS at 30 minutes and from the first to the sixth hour, the 12th hour, and 24 hours postoperatively. Hemodynamic parameters, HR, MAP, adverse effects (nausea, vomiting, drowsiness), time to first rescue analgesia, total analgesic consumption, and overall satisfaction on a 5-point Likert scale were recorded. Rescue analgesia, diclofenac 75 mg IV, was administered for VAS ≥4. If pain persisted after 30 minutes, tramadol 50 mg IV was given. We transferred patients from the PACU after achieving a modified Aldrete score ≥9 [18].

Statistical methods

We performed statistical analyses using SPSS Statistics for Windows, version 17.0 (IBM Corp., Armonk, NY, USA). Continuous variables were presented as mean ± SD, and categorical variables were presented as absolute numbers and percentages. We compared baseline demographic and perioperative variables across groups to assess initial comparability and minimize selection bias. We assessed normality before statistical analysis using the Shapiro-Wilk test. We analyzed normally distributed continuous variables using ANOVA. If the F-value was significant and variances were homogeneous, we used the Tukey multiple-comparison test to assess between-group differences; otherwise, we applied Tamhane’s T2 test. Categorical variables were analyzed using the chi-square test. For all statistical tests, a p-value less than 0.05 was considered statistically significant.

## Results

Demographic characteristics

The study included 75 patients, with 25 in each group. The three groups were comparable in age, sex distribution, BMI, ASA physical status, and duration of surgery, with no statistically significant differences in baseline demographic or perioperative characteristics (p >0.05). There is no missing data. Surgery types in groups C, D, and M were FESS, FESS with septoplasty, septorhinoplasty, FESS with grommet insertion, and rhinoplasty, with no statistically significant differences among them. Table 1 summarizes the demographic and baseline characteristics.

**Table 1:** Demographic and baseline characteristics Pearson’s chi-square test was used for categorical parameters (sex and ASA physical status) to evaluate differences in proportion distribution across groups. The one-way ANOVA (F-test) was used for continuous parameters (height, weight, BMI, and duration of surgery) to evaluate mean differences across the three study groups. Data are expressed as mean ± SD or number (%); a p-value <0.05 is considered statistically significant. ASA: American Society of Anesthesiologists; Group C: Control group; Group D: Dexmedetomidine group; Group M: Magnesium sulfate group

Variable		Group C (n=25)	Group D (n=25)	Group M (n=25)	Test statistic	p-value
Sex	Male	12 (48%)	12 (48%)	13 (52%)	χ² = 0.107	0.94
	Female	13 (52%)	13 (52%)	12 (48%)		
ASA physical status	ASA I	19 (76%)	22 (88%)	18 (72%)	χ² = 2.066	0.356
	ASA II	6 (24%)	3 (12%)	7 (28%)		
Baseline characteristics	Height (cm)	161.92 ± 4.62	163.64 ± 5.24	162.08 ± 5.66	F = 0.838	0.43
	Weight (kg)	60.40 ± 4.19	61.12 ± 4.17	60.16 ± 3.90	F = 0.373	0.69
	BMI (kg/m²)	23.00 ± 1.21	22.83 ± 1.13	22.93 ± 0.80	F = 0.162	0.84
	Duration of surgery (min)	57.20 ± 10.71	57.80 ± 11.64	57.00 ± 8.66	F = 0.040	0.96

Postoperative pain scores

We assessed VAS scores at 30 minutes, the first to sixth hour, the 12th hour, and 24 hours postoperatively. Table 2 presents the VAS scores for the study groups at various time points. Between groups D and M, the VAS scores were comparable at most time points, with a trend toward lower scores in group D during the first few hours. However, the difference was not statistically significant at any interval. By 12 hours and 24 hours, the VAS scores were comparable among all three groups (p >0.05). Group D had significantly lower VAS scores than group C at most early postoperative time points up to six hours (p <0.05). Group M also showed lower VAS scores than group C during the early postoperative period (p <0.05).

**Table 2:** Mean pain scores (VAS) among the study groups Overall group comparison: Intergroup comparisons across all three groups were analyzed using one-way ANOVA, expressed as the F-statistic (degrees of freedom df_1_ = 2, df_2_ = 72). Pairwise comparisons: Post-hoc intergroup pairwise comparisons were performed using the Tukey multiple-comparison test, with test values reported as post-hoc t-statistics and p-values. Statistical significance was set at p < 0.05. VAS: Visual analogue scale; Group C: Control group; Group D: Dexmedetomidine group; Group M: Magnesium sulfate group

Time	Group C (n=25)	Group D (n=25)	Group M (n=25)	Test Statistic	p value	Group C vs. Group D	Group C vs. Group M	Group D vs. Group M
	Mean ± SD					t-value (p-value)		
30 min	3.68 ± 1.8	0.46 ± 0.5	0.68 ± 0.9	F= 56.44	<0.001	9.51(<0.001)	8.86 (<0.001)	0.65 (0.103)
1st hour	3.64 ± 1.19	0.64 ± 0.76	2.04 ± 0.84	F= 62.60	<0.001	11.18 (<0.001)	5.96 (<0.001)	5.22 (<0.001)
2nd hour	3.36 ± 0.91	1.24 ± 0.78	2.60 ± 1.47	F= 24.05	<0.001	6.84 (<0.001)	2.45 (0.043)	4.39 (<0.001)
3rd hour	3.56 ± 0.51	1.88 ± 0.83	2.72 ± 1.24	F= 21.28	<0.001	6.52 (<0.001)	3.26 (0.005)	3.26 (0.007)
4th hour	3.40 ± 0.58	2.08 ± 0.99	2.68 ± 1.03	F= 13.78	<0.001	5.24 (<0.001)	2.86 (0.003)	2.38 (0.041)
5th hour	2.96 ± 0.20	2.32 ± 0.94	2.40 ± 0.71	F= 6.38	0.003	3.28 (0.002)	2.87 (0.016)	0.41 (0.736)
6th hour	2.56 ± 0.58	2.00 ± 0.81	2.24 ± 0.66	F= 4.14	0.021	2.87 (0.007)	1.64 (0.076)	1.23 (0.259)
12th hour	1.36 ± 0.49	1.40 ± 0.5	1.32 ± 0.69	F= 0.12	0.883	0.25 (0.969)	0.25 (0.814)	0.50 (0.641)
24 hours	0.84 ± 0.8	0.72 ± 0.61	0.6 ± 0.58	F= 0.80	0.312	0.63 (0.803)	1.27 (0.420)	0.63 (0.803)

Hemodynamic parameters 

Baseline mean HR was comparable across groups C, D, and M (79.84±8.52, 80.72±6.85, and 79.70±7.81 beats per minute (BPM), respectively; p= 0.883). Similarly, baseline MAP (mmHg) was comparable among the groups (82.08±5.36, 80.68±6.14, and 80.16±4.64; p = 0.435). 

In the postoperative period, group D demonstrated significantly lower HR than group C at PACU admission (72.56±4.10 vs. 84.24±8.06), 30 minutes (65.36±4.19 vs. 96.88±18.52), first hour (64.72±3.87 vs. 99.20±12.22), and second hour (65.20±3.70 vs. 95.08±7.70), with highly significant differences (p <0.001) as shown in Figure 1. Group M also showed lower HR than group C at PACU admission (80.00±5.00 vs. 84.24±8.06; p<0.05) and at 30 minutes (78.32±7.95 vs. 96.88±18.52) and the first hour (81.24±10.59 vs. 99.20±12.22), with very significant differences (p <0.001). However, HR values became comparable among groups beyond two to three hours (p >0.05). Between groups D and M, HR was consistently lower in group D at PACU admission, 30 minutes, first hour, and second hour (p ≤0.001).

**Figure 1:**
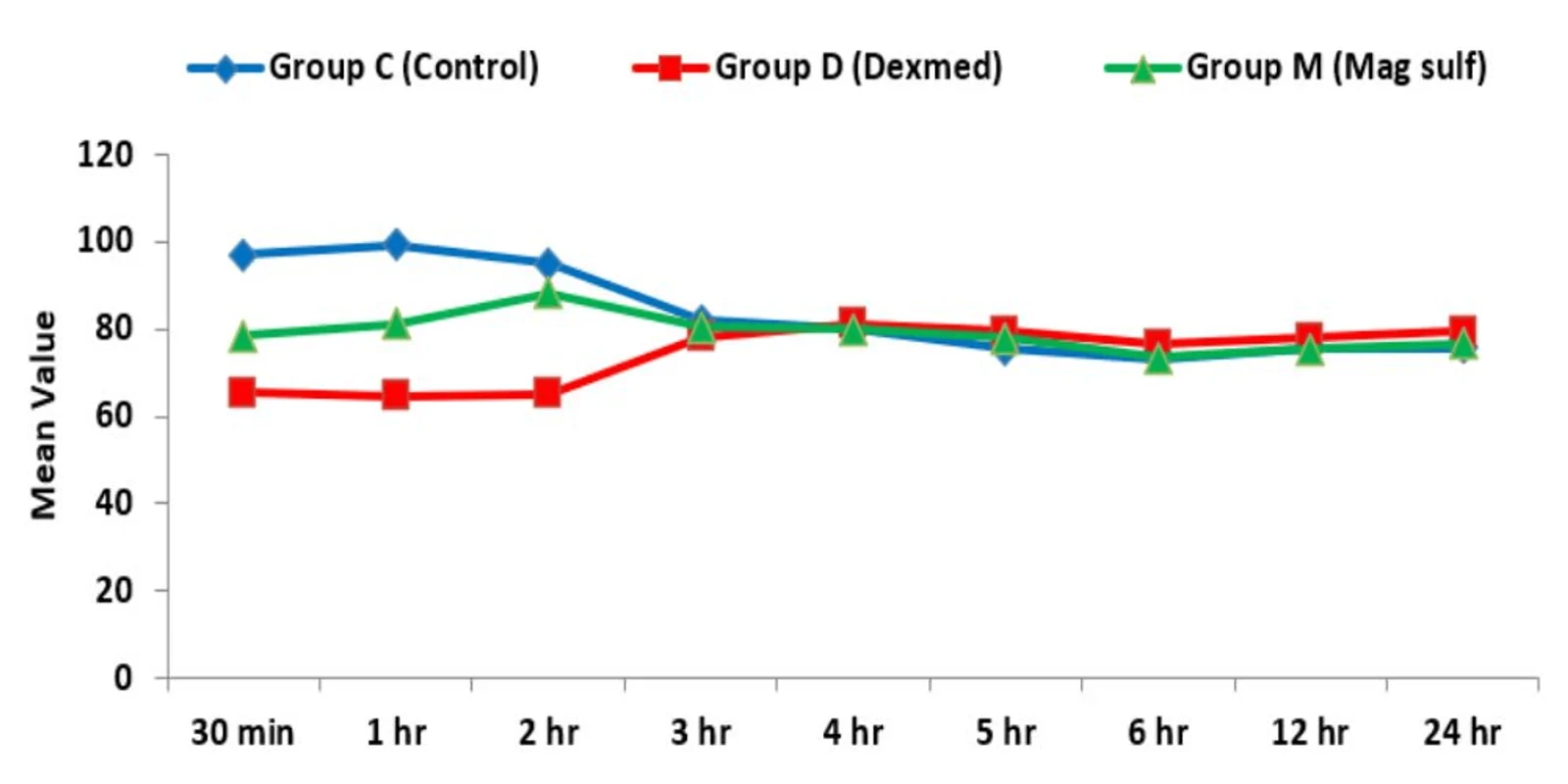
Mean HR in the study groups N=25 in each study group; HR is recorded in BPM; HR: Heart rate, BPM: Beats per minute

For MAP, group D showed significantly lower values than group C at PACU admission (69.8±3.05 vs. 85.84±9.35), 30 minutes (66.6±2.96 vs. 86.12±11.04), first hour (71.8±3.85 vs. 87.28±9.86), and second hour (69.8±3.01 vs 87.48±6.39), with highly significant differences (p <0.001), as depicted in Figure 2. Group M also had lower MAP than group C at PACU admission (73.24±4.52 vs. 85.84±9.35) and at 30 minutes (72.20±5.09 vs. 86.12±11.04), both statistically significant (p <0.001). The MAP was lower in group D than in group M at PACU admission (69.8±3.05 vs. 73.24±4.52; p<0.05) and at 30 minutes (66.6±2.96 vs. 72.20±5.09; p<0.05). In the first hour (71.8±3.85 vs. 84.36±6.90) and second hour (69.8±3.01 vs. 81.84±4.89), group D continued to show lower MAP with highly significant differences (p <0.001). No significant differences in MAP were observed among groups beyond three hours postoperatively (p >0.05).

**Figure 2:**
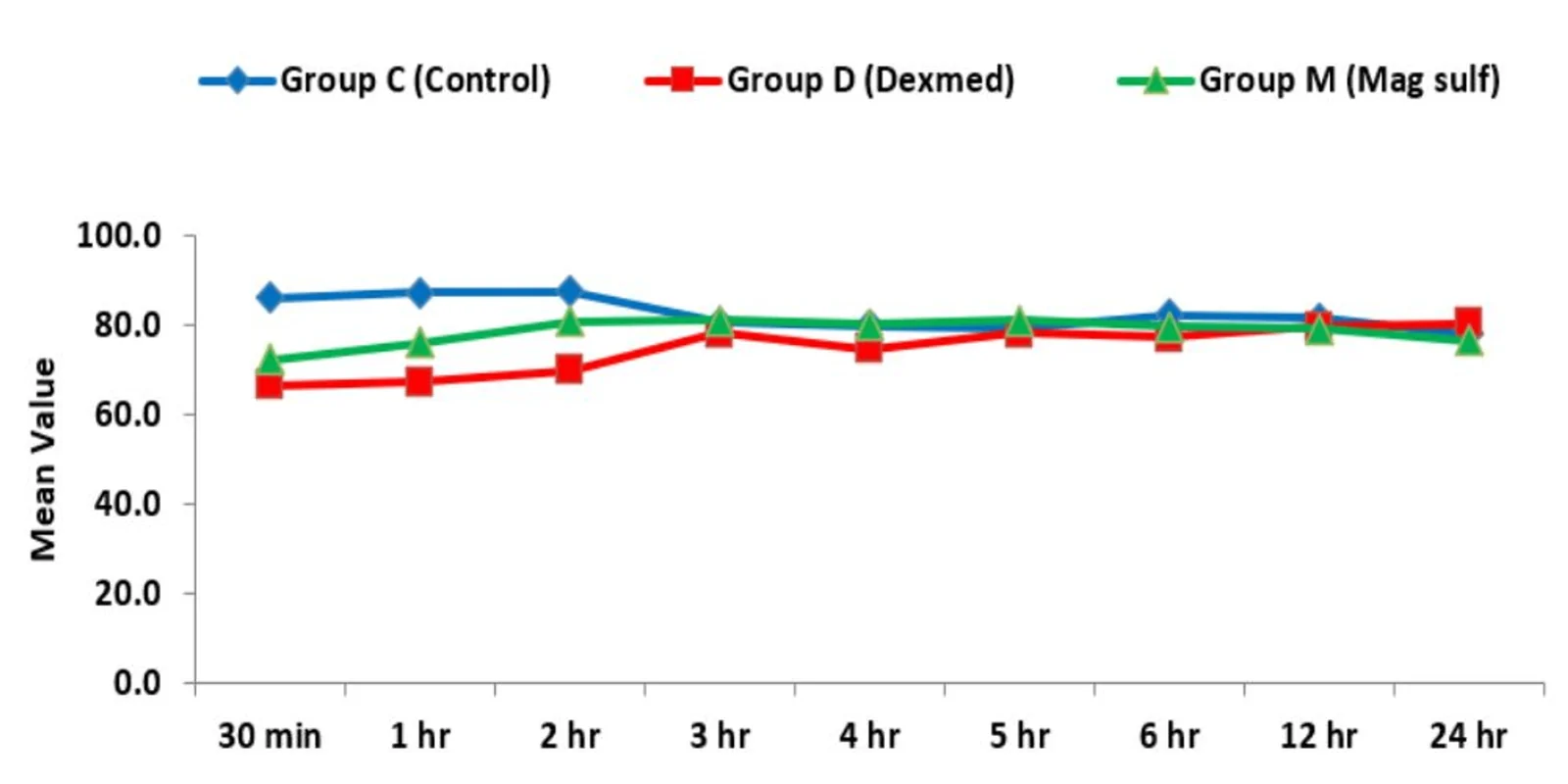
Mean MAP in the study groups N= 25 in each group; MAP: Mean arterial pressure in mmHg.

Rescue analgesic requirement

The incidence of patients requiring first rescue analgesia (diclofenac) differed significantly across groups: 24 out of 25 (96%) in group C, 11 out of 25 (44%) in group M, and eight out of 25 (32%) in group D. Among those requiring rescue medication, the mean time to first rescue analgesia was significantly prolonged in group D (285.00±46.63 min, n = 8) and group M (147.27±49.21 min, n = 11) compared to group C (56.25±39.87 min, n = 24; p < 0.001), as depicted in Table 3. Similarly, 23 out of 25 (92%) patients in group C required second rescue analgesia (tramadol), compared with five (20%) in group D and 3 (12%) in group M.

**Table 3:** Time to rescue analgesics Overall intergroup comparison was analyzed using one-way ANOVA across the three study groups. The test statistic is reported as the ANOVA F-value. Pairwise Intergroup Comparisons: Post-hoc pairwise comparisons between individual groups (group C vs. group D, group C vs. group M, group D vs. group M) were performed using the Tukey multiple-comparison test, and test statistics for pairwise comparisons are reported as post-hoc t-values alongside their respective p-values. Significance level: A p-value of < 0.05 was considered statistically significant. The total enrolled sample size was n = 25 per group. Reported sample sizes (n) for the time to rescue analgesia vary, as the calculations only include patients who reached the pain score threshold requiring rescue medication.

Rescue analgesia	Group C	Group D	Group M	F-value (p- value)	Group C vs. Group D	Group C vs. Group M	Group D vs. Group M
	Mean ± SD				t-value (p-value)		
Time to first rescue analgesic (mins) diclofenac 75mg IV	56.25 ± 39.87 (n=24)	285± 46.63 (n=8)	147.27 ±49.21 (n=11)	85.09 (<0.001)	12.85 (<0.001)	5.74 (<0.001)	6.80 (<0.001)
Time to second rescue analgesic (mins) tramadol 50mg IV	208.69 ± 47.41 (n=23)	365.90 ± 35.72 (n=5)	260.0 ± 34.64 (n=3)	11.65 (<0.001)	4.71 (<0.001)	1.88 (0.071)	3.31 (0.003)

In group C, one (4%) patient received no analgesic, one (4%) received one dose, and 23 (92%) received two doses. In group D, 12 (48%) received no analgesic, eight (32%) received one dose, and five (20%) required a second dose. In group M, 14 (56%) received no analgesic, eight (32%) received one dose, and three (12%) required a second dose. Total rescue analgesic consumption was lowest in group D, followed by groups M and C, with a significant difference (p <0.001), as shown in Table 4. 

**Table 4:** Mean total rescue analgesic consumption in the study groups Data are presented as observed counts and percentages, i.e., n (%). Chi-value: Pearson chi-Square statistic (d*f* = 4). Statistical significance was defined as p <0.05. A statistically significant association was observed between the group allocation and the total number of rescue analgesic doses required (p < 0.001).

Total rescue analgesic doses	Frequency (%)			Test statistic	p-value
	Group C	Group D	Group M		
0	1 (4%)	12 (48%)	14 (56%)	χ² =55.41	<0.001
1	1 (4%)	8 (32%)	8 (32%)		
2	23 (92%)	5 (20%)	3 (12%)		
Total	25	25	25		

Adverse events

All patients were monitored in the PACU with standard parameters. Although postoperative HR and MAP were lower in groups D and M than at baseline, no patient required medical intervention in the PACU. No cases of prolonged sedation were observed in group D, and no neuromuscular weakness or delayed recovery was noted in group M. All patients achieved a modified Aldrete score ≥9 and were shifted to the ward. No serious adverse events were reported during the 24-hour follow-up.

Patient satisfaction

Around 12% of the patients in group D (n=3) had a fair (score 3) experience, 32% (n=8) had a good (score 4) experience, and 56% (n=14) had an excellent (score 5) experience on the 5-point Likert scale for overall patient satisfaction. In group M, 4% of patients (n=1) reported a poor experience (score 2), 28% (n=7) a fair experience (score 3), 52% (n=13) a good experience (score 4), and 16% (n=4) an excellent experience (score 5). In group C, 36% of patients (n=9) rated their experience as poor (score 2), 52% (n=13) as fair (score 3), 12% (n=3) as good (score 4), and none rated it excellent (score 5). Group D patients had a better satisfaction score, followed by groups M and C (p <0.001).

## Discussion

This prospective comparative study compared the effects of pre-emptive dexmedetomidine and magnesium sulfate on postoperative pain relief in patients undergoing nasal surgery under general anesthesia. The study included 75 patients across three groups: (1) dexmedetomidine (group D), magnesium sulfate (group M), and control (group C). Baseline demographic variables, ASA status, duration, and type of surgery were comparable across the three groups, suggesting clinical similarity at baseline.

We assessed postoperative pain using the VAS score from 30 minutes to 24 hours. Pain scores in the dexmedetomidine group were significantly lower than those in the control group through the fourth postoperative hour, with clinically significant differences persisting through the sixth hour. Although both dexmedetomidine and magnesium sulfate demonstrated lower VAS scores than control at 30 minutes, there was no statistically significant difference between the two active drugs at that time point. Therefore, both agents effectively attenuated immediate postoperative pain. However, from the first to the fourth postoperative hour, dexmedetomidine consistently showed lower pain scores than magnesium sulfate. Beyond the fifth hour, the differences between the two drugs were no longer significant, likely because most patients had already received rescue analgesics, and overall pain intensity in nasal surgeries tends to decline after the early postoperative period.

Our findings are broadly consistent with previous studies. Kim et al. demonstrated improved postoperative pain scores with dexmedetomidine infusion up to 24 hours [16]. The longer duration of analgesia in their study may reflect differences in dosing strategy and infusion duration. Similarly, Song et al. reported prolonged time to first rescue analgesia and reduced opioid consumption with dexmedetomidine after craniotomy [19]. Although the surgical models differ in intensity, the overall trend of enhanced early postoperative analgesia aligns with our observations. Other investigators, such as Goksu et al. and Hwang et al., also reported improved analgesia with dexmedetomidine [20,21]. However, the duration of effect varied, depending on the surgical procedure and the use of postoperative opioids. This variability likely reflects differences in surgical pain severity, dosing regimens, and adjunct analgesic protocols.

Regarding magnesium sulfate, our study demonstrated improved analgesia compared with patients who did not receive any pre-emptive analgesic adjuvant (group C), particularly during the first postoperative hour and up to five hours. These findings are comparable to those of Elsersy et al. and Cizmeci & Ozkose, who reported reduced early postoperative pain after FESS and septorhinoplasty, respectively [22,23]. However, some studies in major abdominal or spinal surgeries have demonstrated more prolonged analgesic effects with magnesium. The relatively shorter duration observed in our study may be explained by the mild-to-moderate nature of postoperative pain following nasal procedures [9,17].

Dexmedetomidine was associated with lower HR and MAP in the early postoperative period compared to magnesium sulfate and the control group. These differences were most evident during the first two postoperative hours. Previous studies on nasal or sinus surgeries have used loading and maintenance doses of dexmedetomidine to achieve controlled hypotension [11-13]. But we intended to use a dose to provide postoperative analgesia and fewer hemodynamic changes. The reductions remained within clinically acceptable limits, and no patient required pharmacological intervention. These findings are consistent with studies by Rokhtabnak et al., Goksu et al., and Olutoye et al., who also reported lower heart rates in dexmedetomidine groups during early recovery [13,20,24]. Magnesium sulfate similarly reduced HR compared with control, though the effect was less pronounced than with dexmedetomidine. The early hemodynamic stability observed may be attributed to dexmedetomidine's sympatholytic properties and magnesium sulfate's vasodilatory effects.

The time to first rescue analgesic was significantly prolonged in the dexmedetomidine group (285 ± 46 minutes), followed by magnesium sulfate (147.27 ± 49.21 minutes), and was shortest in the control group (56.25 ± 39.87 minutes). This finding supports the properties of the opioid-sparing effect and prolonged analgesia of dexmedetomidine reported in previous literature [19,21]. In the dexmedetomidine group, eight patients required rescue analgesia, with five needing a second dose. Additionally, effective pain management and a lower need for rescue analgesia may have contributed to the higher overall satisfaction reported in our study.

In the magnesium sulfate group, 11 patients required the initial rescue analgesic dose, of whom three required additional doses, whereas 24 patients in the control group required rescue analgesia. The lower need for postoperative rescue analgesia with magnesium sulfate is consistent with findings from Kiran et al. and Elsersy et al., who reported reduced postoperative analgesic consumption with magnesium sulfate [7,22]. 

Interpretation

Pre-emptive dexmedetomidine has provided lower postoperative pain scores and minimized the need for rescue analgesia when compared with magnesium sulfate and control. The analgesic effect was most pronounced in the early postoperative period, alleviating discomfort associated with nasal packing. Magnesium sulfate also provided a modest analgesic benefit over control. These findings highlight the importance of non-opioid medications for pain management and compare the two commonly used drugs in nasal surgery, which provide controlled hypotension as well as analgesia at the same time.

Strengths and limitations

First, the prospective design and consecutive enrollment enabled systematic collection of perioperative and postoperative outcomes. Second, blinded outcome assessment minimized potential observer and assessment bias. Third, including a control group and directly comparing two commonly used adjuvants provided clinically relevant comparative data. Predefined eligibility criteria and standardized outcome measures also ensured consistency in patient selection and outcome assessment.

The single-center design, relatively small sample size, and restriction to nasal surgery may limit generalizability. The non-randomized allocation may have introduced allocation bias and residual confounding despite comparable baseline characteristics. Evaluating only a single dose and regimen precluded assessment of dose-response effects, and the study did not assess longer-term outcomes, patient satisfaction, or cost-effectiveness. Future randomized, multicenter studies involving larger sample sizes, different dosing strategies, and broader surgical populations can help to strengthen and validate these observations.

## Conclusions

Dexmedetomidine and magnesium sulfate are well-known agents for providing controlled hypotension during nasal surgery. This prospective, non-randomized comparative observational study demonstrated that both adjuvants also provide postoperative analgesia. Dexmedetomidine was associated with longer analgesia and reduced rescue analgesic requirements compared to magnesium sulfate. Further, adequately powered, randomized controlled trials are needed to confirm these findings.
